# Operational evaluation of the earlobe arterialized blood collector in critically ill patients

**DOI:** 10.1186/s13728-015-0025-x

**Published:** 2015-04-02

**Authors:** Sergi Vaquer, Jordi Masip, Gisela Gili, Gemma Gomà, Joan Carles Oliva, Alexandre Frechette, Simon Evetts, Thais Russomano, Antonio Artigas

**Affiliations:** Critical Care Centre, Corporació Sanitària Universitària Parc Taulí, Universitat Autònoma de Barcelona, CIBER Enfermedades Respiratorias, Sabadell, Spain; Science, Technology & Engineering, Wyle GmbH, Cologne, Germany; MicroG Centre-PUCRS, Porto Alegre, Brazil; CHAPS, King’s College, London, UK; Servei de Medicina Intensiva, Corporació Sanitària Parc Taulí, Parc Taulí 1, 08208 Sabadell, Spain

**Keywords:** Acute respiratory failure, Space medicine, Capillary blood, Intensive care medicine, Mechanical ventilation

## Abstract

**Background:**

The new Earlobe Arterialized Blood Collector (EABC®) is a minimally invasive prototype system able to perform capillary blood collection from the earlobe (EL) with minimal training and risk. This system could improve medical emergency management in extreme environments. Consequently, a prospective validation study was designed to evaluate operational performance of the EABC® in a cohort of critically ill patients.

**Methods:**

Arterialized capillary blood was sampled from the EL of 55 invasively ventilated patients using the EABC® following a validated procedure. Operational characteristics such as the number of cuts and cartridges required, sampling failure/success ratio, bleeding complications, storage requirements and other auxiliary aspects were recorded. Result turnaround laboratory times (TAT) were compared with published references.

**Results:**

Blood collection was as easily performed on one earlobe as the other. Twenty-six minutes (mean 25.8; SD = 3.8) were required to obtain results, 15 min for patient preparation (mean 15.3; SD = 2.6) + 11 min for sampling and analysis (mean 11.4; SD = 2.1), which is similar to published hospital reference laboratory TAT. The average number of cartridges required was 1.3 (1–3; mode = 1) with the mean number of cut attempts being 1.2 (1–4; mode = 1). Problems/difficulties occurred in 59% of cases but were mainly attributed to patient’s demographic characteristics, with only 10% attributable to the collector (superficial cut, blood leak, collector misalignment and obstructed vision). Haemostasis was quickly achieved with minimum complications. Storage of the complete sampling kit required a 300 × 300 × 300 mm box. Two 9-V batteries were used during the 2-year study period.

**Conclusions:**

The new EABC® system concept is safe, fast and easy to use. Observed problems/difficulties are easily amendable with certain design modifications. Definitive versions of the prototype have the potential for significant benefits for isolated and extreme environments in medicine.

## Background

Critical medical situations are likely to occur during space missions in the future [1]. In such situations, blood analysis capabilities, and especially arterial blood gasometrical evaluation, will be essential for proper medical management. Earlobe arterialized capillary blood gas analysis has been proposed as an adequate substitute for some arterial measures. This technique is based on achieving a capillary arterio-venous shunt in the earlobe so that capillary blood gas content approximates arterial levels [2]. Nevertheless, discordant results are found in the literature on the accuracy of this technique, which has been extensively evaluated in healthy volunteers during exercise [3,4], in patients with different forms of respiratory failure [5-9], in patients under mechanical ventilation [10] and more recently in a meta-analysis [11].

Performance of a new prototype system for estimating arterial gas content from earlobe capillary arterialized blood, the Earlobe Arterialized Blood Collector (EABC®) + i-STAT® portable gas analyser, has been evaluated in healthy volunteers during head venous congestion, hypoxia and microgravity [12,13] and in patients with chronic renal failure [14]. This new equipment minimizes room air contamination and enables standardization of sampling procedures. Results show good concordance of measures with arterial samples and much better safety profile, training requirements and patient tolerance than arterial sampling. It has not, however, been tested in critically ill patients. Therefore, a validation study was designed to evaluate its analytical performance and its operational requirements in a cohort of critically ill patients admitted to a general intensive care unit (ICU) for various conditions. Results from the analytical performance evaluation evidenced that the EABC® + i-STAT® is precise, capable of detecting extreme gasometric values in critically ill patients and could permit diagnosis and classification of patients presenting with acute respiratory distress syndrome (ARDS) [15]. We present hereafter the operational evaluation of this equipment in the routine healthcare of critically ill patients.

## Methods

Operational performance of the EABC® (Microgravity Centre/FENG-PUCRS, Brazil) + i-STAT® portable analyser (Abbot, United States of America) was evaluated in a cohort of adult patients admitted to a general ICU. Common demographic and clinical variables of study patients were recorded.

Two operators were trained on the technique and performed all evaluations. Training consisted of two separated workshops in which the producer presented the device and main procedures. Additional reference material and remote support were available as required after the practical sessions.

Informed consent by the patient or next of kin was required and local ethics committee accepted the study protocol (IRB: 2009593. Comité Ètic d’Investigació Clínica, Corporació Sanitària Universitària Parc Taulí, Sabadell, Spain).

**Figure 1:**
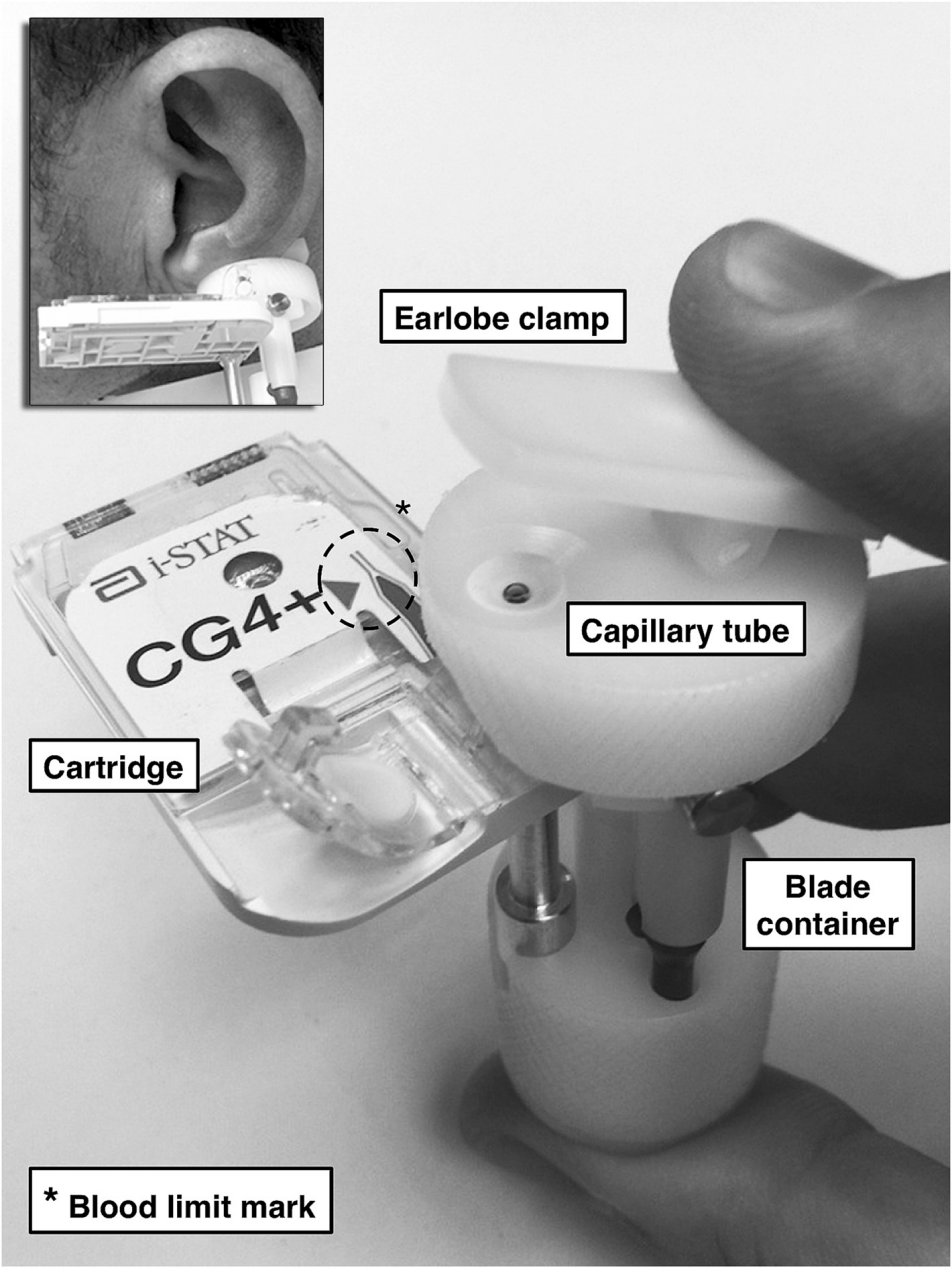
**The Earlobe Arterialized Blood Collector (EABC®).**

The sampling procedure consisted of three phases: preparatory, blood collection or sampling and post-sampling processes. Problems and difficulties observed during each phase were recorded. The time expenditure from decision to sample to the completion of post-sampling procedures was measured (turnaround time (TAT)). Other auxiliary aspects, such as calibration, power consumption, software update and storage requirements were also taken into account. The preparatory phase consisted of analyser initialization, sampling equipment preparation, local area setup, assessment of patient readiness for sampling, earlobe selection, cleaning/disinfection and capillary arterialization. Vasodilation cream (2% nitro-glycerine cream) and earlobe massage were used for achieving capillary arterialization. In the blood collection phase, a blood sample was collected anaerobically (i.e. without contact with air) using the EABC® (Figure 1). The collector performed a minor cut on the earlobe and aligned the blood sample with an electronic analysis cartridge which was subsequently read by the i-STAT® portable analyser. This device has already been used in conjunction with the EABC® in previous validation studies due to its appropriate performance advantages, such as accuracy, precision, portability and rapidness of results [16,12]. The results were displayed on the analyser screen and were stored in its internal memory. This information can be printed or synchronized with a computer if required. The number of cuts and cartridges required for obtaining a valid sample, and the overall sampling failure/success ratio, were recorded. Post-sampling procedures comprised achievement of haemostasis, earlobe cleaning, shutdown of equipment, storage and addressing delayed complications. Since the final version of the EABC® was intended to be totally disposable, time and problems observed during prototype assembly and sterilization after use are not presented in the current evaluation but were reported to the producer for design optimization. Patients were grouped chronologically and the sampling failure/success ratio was analysed in order to evaluate the learning curve of the procedure.

Data were analysed using SPSS version 19 statistical software (International Business Machines. Armonk, New York. United States of America) and is presented as common statistical parameters (ratio, percentage, mean, mode and SD). Chi-Square test was used for evaluating qualitative variables. Two-tailed significance threshold was established at *p* < 0.05 for all statistical tests, and the 95% confidence interval is provided where available.

## Results

During this study, 55 capillary blood collections were performed on 55 critically ill patients and the results were evaluated. A brief summary of patient characteristics can be found in Table 1.

**Table 1:** **Study patient characteristics**

Patients	55
Male	40 (72.7%)
Age	63 (24–83)
Arterial hypertension	23 (41.8%)
Diabetes mellitus	19 (34.5%)
Chronic cardiac failure	4 (7.3%)
Severe vasculopathy	4 (7.3%)
Renal insufficiency	5 (9.1%)
Diagnostic at ICU admission	
Severe sepsis	21 (38.1%)
Respiratory failure	7 (12.7%)
Severe trauma	7 (12.7%)
Neurological	6 (10.8%)
Cardiogenic shock	5 (9.1%)
Miscellaneous	9 (16%)

The mean total time from decision to sample to results was 26 min (mean 25.8; SD = 3.8). Fifteen minutes (mean 15.3; SD = 2.6) were required for the preparatory phase and 11 min (mean 11.4; SD = 2.1) for blood collection and post-sampling procedures. The i-STAT® portable analyser required one automated calibration per sampling day which required 2–3 min.

**Figure 2:**
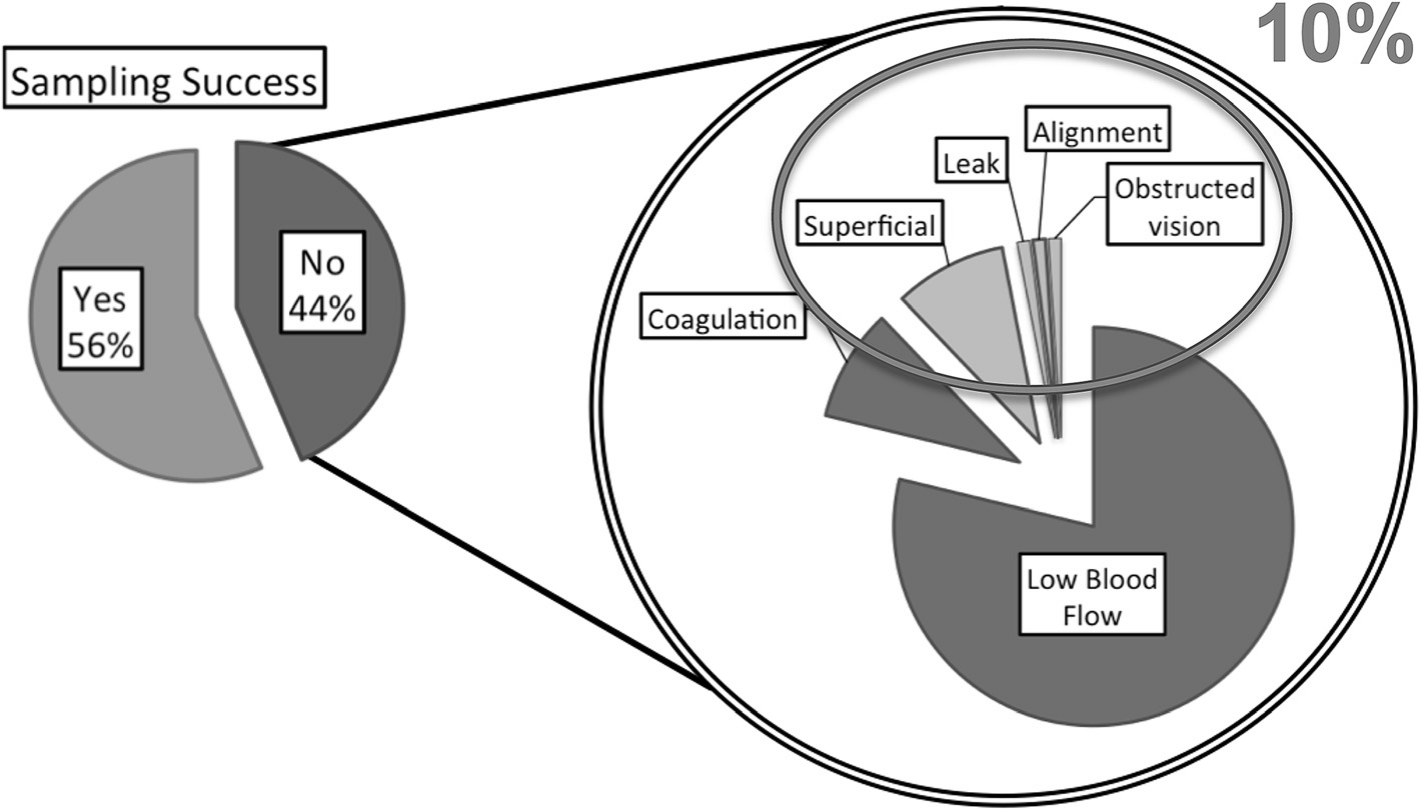
**Sampling failure and its causes.**

Samples of earlobe (EL) capillary blood were obtained in 56.4% of the cases. Sampling problems attributed to the blood collector included insufficient depth of cut (9%), blood leak, collector misalignment and investigator-obstructed vision. Other problems not attributed to the collector such as low-blood flow from the EL incision and blood coagulation before reaching the cartridge were observed in 78.1% and 9% of the cases, respectively (Figure 2). Whenever blood collection was achieved, it was performed as easily on one EL as the other.

**Figure 3:**
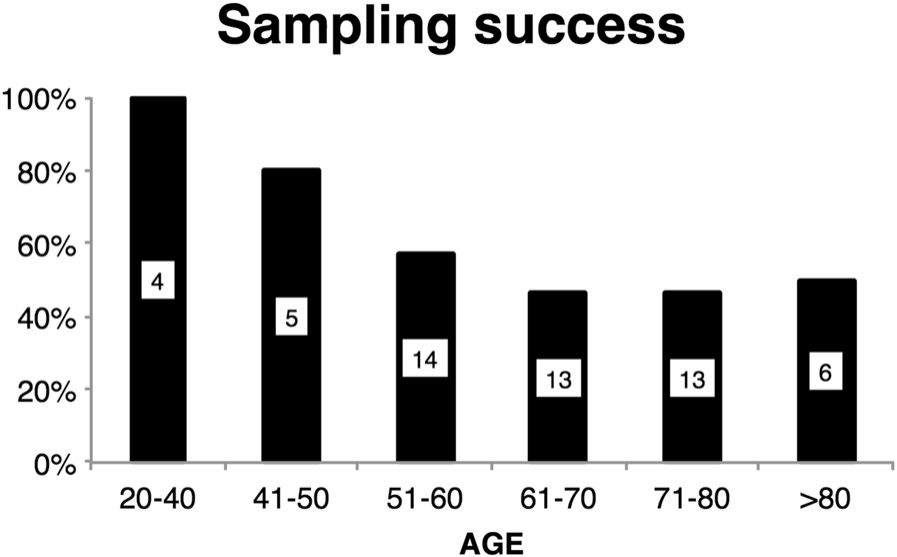
**Sampling success according to patient age.**

The success/failure ratio was calculated chronologically as a rolling figure throughout the study. Results indicate that 35 sampling attempts may be required before the ratio stabilizes; however, an examination of patient characteristics in relation to this ratio showed that older patient clusters were associated with less favourable success/failure ratios. The effect of age in sample success could be observed when this ratio was analysed in different age groups (Figure 3), e.g. patients <50 years (*n* = 9) exhibited a sampling success of 90%. Additionally, the rather slow-inclusion rate meant that experience was slowly gained, which could have affected the observed learning curve.

EL arterialization with vasodilation cream and EL massage was performed during 2.5 min for the first 20 patients but was later increased to 5 min for the remaining 35 patients of the study. Earlobe blood congestion was achieved in both cases. Increasing arterialization time did not affect sampling success rate (OR = 0.86; *p* = 0.8; CI 95% = 0.28–2.67).

The average number cartridges required to obtain a measure was 1.3 (range = 1–3; mode = 1) and associated with a mean of 1.3 cut attempts (range = 1–4; mode = 1). Haemostasis was easily achieved with short-bleeding times (92.7% < 10 min) without complications (one case of suspected infection that quickly resolved spontaneously). Furthermore, no operator injuries or contamination by contact with patient’s blood were reported.

Auxiliary procedures for the i-STAT® portable analyser are described hereafter. Power consumption was low requiring one battery change (2 × 9-V battery) during the 2-year period of the study. Regular software updates were undertaken as required by the manufacturer. No additional auxiliary i-STAT® equipment was required for this study. Details on other i-STAT® technical specifications and performance characteristics can be found in the producer manual and are available online [16].

Storage space required for the i-STAT® portable analyser, automatic calibration system, computer-synchronization device, ten units of the EABC® and additional material for ten blood collections (sterile gloves, cleaning gauzes, etc.) fitted into a 300 × 300 × 300 mm box.

Cartridge shelf life if stored at room temperature (18°C–30°C) was 2 months; however, storage at 8°C increased lifetime until stipulated expiration date (minimum of 6 months).

## Discussion

The results from the present operational evaluation demonstrate that the EABC® + i-STAT® system concept is safe, fast and easy to use. The observed problems/difficulties attributable to the collector accounted for 10% of the cases and comprised of superficial cut, blood leak, collector misalignment and operator’s obstructed vision. These would be easily amendable with certain design modifications.

The results from the analytical performance evaluation have been published elsewhere [15] and showed good precision levels (coefficient of variation < 10% in all gasometrical variables) and enough accuracy to detect extreme gasometrical alterations of the EABC® + i-STAT® system (percentage of error < 20% in all gasometrical variables) when compared with direct arterial reference samples. This new system was also able to correctly classify ARDS patients according to severity (sensitivity = 100%, specificity = 92.3%, kappa coefficient = 0.85) [15]. Therefore, the new technique was considered useful in the initial diagnosis and treatment of medical emergencies and could prove to be valuable if implemented in a medical protocol as a first evaluation tool of critically ill patients in remote environments. From an operational point of view, a limited sampling success rate was observed. The main causes of sampling failure were low-blood flow from the earlobe and blood coagulation, which had not been evidenced in previous validation studies using the EABC® + i-STAT® system [14,12,13] or when other capillary blood collection methods were used [3-10]. A separate evaluation was performed to ascertain what factors could influence earlobe capillary blood flow and affect sampling procedure, results of which have been published elsewhere [15]. Using a multivariate analysis, which took into consideration several patient demographics and medical characteristics, the results evidenced that patient age was the only variable associated with sampling failure. It was hypothesized that microvascular ageing of the earlobe capillary bed could have influenced blood flow and blood delivery to the cartridge, leading to the observed low-sampling success ratio in older patients [15]. Nevertheless, success rate was 90% in patients <50 years (*n* = 9) and 100% in 40-year-old patients (*n* = 4), which would support the use of the EABC® + i-STAT® system in a wide range of patients, especially those in isolated and extreme environments, such as astronauts in space [17] or explorers of remote locations on Earth.

The training required for learning the procedure of earlobe arterialized capillary blood sampling with the new EABC® + i-STAT® system was short, requiring only two workshops. Although a learning curve may exist, its interpretation was difficult and unreliable due to clustered patient distribution and was not used to determine training needs of the procedure. The two operators involved in the study reported that the sampling procedures were easy to perform in either of the patient’s earlobes. Furthermore, the system was found to be portable, manageable and efficient, requiring a reduced number of cuts and analysis cartridges in most of the cases. These findings confirm that the new system is easy to use and requires minimum training as reported in previous studies.

TAT is a key determinant of laboratory performance evaluation from an operational point of view [18]. In the present study, a practical approach was chosen and TATs were calculated from the decision to sample until the finalization of post-sampling procedures. This estimation of TAT was considered to be the best interpretation and information of the real operational needs of the technique. Results showed that TAT of the whole procedure was 26 min, lying below current TATs of most tertiary hospitals [18]. These results were considered adequate given the specific characteristics of the medical situations where the procedure could be used. However, since TAT calculation can be controversial sometimes [18], direct analysis time (or isolated laboratory TAT) can be evaluated. In this context, if preparatory procedures are excluded, results from blood gas analysis can be quickly obtained in 120 s using the i-STAT® portable analyser, which still represents an appropriate time to results [16]. Furthermore, since earlobe arterialization was achieved in spite of increases in vasodilation time, further decrease in time expenditure for the whole procedure can be envisaged and could lead to a theoretical reduction of TAT to 23.5 min. Additionally, auxiliary procedures were found to require minimum time expenditure.

This study also confirmed the safety of the new system for both the patient and the operator. There was only one case of suspected infection of the EL, which resolved spontaneously. No episodes of accidental injury with the cutting blade or contamination of the operator with patient’s blood occurred. The safe use of the device was possible due to the containment of both blood and blade within the collector during operation. These findings are in agreement with previous studies in which no complications were reported.

The equipment utilized for providing autonomous gasometrical analysis capabilities was found to require a small storage volume. Furthermore, although the shelf life of a refrigerated cartridge can be extended to several months, room temperature storage during a maximum of 2 months is also possible. These findings make this new system very suitable for environments with limited storage capabilities such as the International Space Station and other space systems, as well as in extreme and isolated environments on Earth.

The present study has a number of limitations. First, the evaluation of the usability of the proposed new technique and collector comes substantially from the subjective opinion of operators, which could induce a bias in the evaluation. However, both operators were able to learn the procedure quickly and required only one cut to obtain a valid blood sample in most of the cases, which supports that sufficient skills on the use of the technique had been achieved. Second, as previously discussed, the presence of a learning curve may suggest that 35 sampling attempts are required to learn the procedure. However, unbalanced distribution of patients makes this assessment unreliable. There must be a minimum number of test rounds required; however, data are insufficient to determine such a number with precision. Third, this evaluation focuses on critically ill patients in the controlled environment of an ICU. While the use of a varied population of such patients may provide useful information that could be transferrable to other similar patients in extreme or remote environments, other uncontrolled factors such as temperature, humidity or atmospheric pressure may influence performance and operational characteristics of the technique. Therefore, additional field studies are required to confirm the potential benefits for patient healthcare in remote environments suggested in the present analysis.

## Conclusions

In conclusion, this evaluation has demonstrated that operational characteristics of the new EABC® + i-STAT® system such as efficiency, sampling performance in specific patients, usability, minimum training requirements and reduced time to results, make it very suitable for use in remote and isolated environments such as space. Good accuracy and diagnostic capabilities in combination with abovementioned operational features suggest that definitive versions of the prototype can offer significant benefits for patient health care in space and other extreme environments. Future studies should focus on confirming the benefits of use of this new equipment and technique in such medical scenarios.

## Abbreviations

EABC: Earlobe arterialized blood collector
ICU: Intensive care unit
ARDS: Acute respiratory distress syndrome
EL: Earlobe
TAT: Turnaround time
